# Refining absolute GFR estimation in children: development and validation of the GROW-GFR equation

**DOI:** 10.3389/fphar.2026.1855031

**Published:** 2026-10-22

**Authors:** Kiera H. Harwood, Stephen Duffull, Christian Davey, Catherine Quinlan, Thomas Forbes, Peter Francis, Tony Lai, Sarath Ranganathan, Amanda Gwee

**Affiliations:** 1 Department of Paediatrics, The University of Melbourne, Parkville, VIC, Australia; 2 Antimicrobials Group, Murdoch Children’s Research Institute, Parkville, VIC, Australia; 3 Certara, Princeton, NJ, United States; 4 School of Mathematics and Statistics, The University of Melbourne, Parkville, VIC, Australia; 5 Kidney Regeneration, Murdoch Research Institute, Parkville, VIC, Australia; 6 Department of Paediatric Nephrology, The Royal Children’s Hospital, Parkville, VIC, Australia; 7 Department of Medical Imaging, The Royal Children’s Hospital Melbourne, Parkville, VIC, Australia; 8 Department of Pharmacy, The Children’s Hospital at Westmead, Sydney, NSW, Australia; 9 Sydney Pharmacy School, Faculty of Medicine and Health, The University of Sydney, Sydney, NSW, Australia; 10 Department of Respiratory and Sleep Medicine, The Royal Children’s Hospital, Parkville, VIC, Australia; 11 Respiratory Diseases Group, Murdoch Children’s Research Institute, Parkville, VIC, Australia; 12 Department of General Medicine and Infectious Diseases, Royal Children’s Hospital, Parkville, VIC, Australia

**Keywords:** antimicrobial, drug dosing, kidney function, paediatrics, pharmacokinetics

## Abstract

**Background:**

Accurate estimation of glomerular filtration rate (GFR) is essential when dosing many medications. Existing paediatric GFR equations vary in accuracy, with only one (Modified Schwartz) validated in children <2 years. This study developed a new paediatric GFR equation (GROW-GFR) using a tobramycin population pharmacokinetic model and evaluated its performance against current equations and nuclear medicine (NM)-GFR scans.

**Methods:**

Retrospective analysis of NM-GFR scans from July 2016-November 2023 in children aged 0–19 years at The Royal Children’s Hospital, Melbourne. Results were included if NM-GFR was ≤150 mL/min and height, weight and serum creatinine were recorded. Accuracy of GFR estimates within 30% and 10% of NM-GFR results (p30, p10 accuracy) and bias for the GROW-GFR, Modified Schwartz, Chronic Kidney Disease in Children Under 25 years (CKiD-U25), Full Age Spectrum (FAS), and FAS-height (HT) equations were assessed.

**Results:**

Overall, 1560 NM-GFR results from 711 children (median age 8.5 years; range 0.1–19) were included (median NM-GFR 55 mL/min; range 0.5–150). GROW-GFR showed a p30 accuracy of 76% and bias of −1.9 mL/min, with consistent performance across all ages. The Modified Schwartz equation had a p30 accuracy of 46% and bias of 17.3 mL/min. In children ≥2 years, GROW-GFR outperformed other equations (p30: 76% vs. 49%–58%; bias −1.9 vs. 13.7–18.4 mL/min). After applying linear mixed-effects models, GROW-GFR p30 accuracy and bias were 80% and −2.5 mL/min, outperforming other equations (p < 0.001).

**Conclusion:**

The GROW-GFR equation provided more accurate GFR estimates across all paediatric ages, including children <2 years, compared to existing equations. Therefore, should be considered the preferred GFR estimation method.

## 1 Introduction

The accurate determination of kidney function, as measured by glomerular filtration rate (GFR), is critical for appropriate dosing of drugs cleared by the kidneys, including several nephrotoxic chemotherapy and antimicrobial agents (Mian and Schwartz, 2017). Although nuclear medicine (NM)-GFR scans are often used to accurately measure GFR, they require the injection of an exogenous, and often radioactive filtration marker (e.g. ^51^Cr-EDTA, ^99m^Tc-DTPA) and the collection of multiple blood and/or urine samples, which can be challenging in children (Stevens et al., 2006; Rahn et al., 1999). Thus, guidelines recommend that these scans be performed only in specific clinical circumstances, such as for patients with malnutrition, heart failure, or cancer, where precise GFR measurements are crucial. Notably, these indications do not extend to dosing guidance for widely used renally cleared agents such as aminoglycosides, despite their substantial global burden of drug-related adverse effects (Prasad et al., 2024; Oun et al., 2018; Hartmann and Lipp, 2003; Kidney Disease: Improving Global Outcomes CKD Work Group, 2024). Therefore, there is a demand among children for reliable GFR equations to estimate kidney function in these patients accurately.

In adults, several creatinine-based equations, such as CKD-EPI and Cockroft-Gault, are used in clinical practice and are integrated in multiple tools to aid drug dosing (Cockcroft and Gault, 1976; Levey et al., 2006; Levey et al., 2009). When these equations are used in paediatric populations, however, their accuracy has been questioned. Multiple studies assessing the performance of adult GFR equations in children have demonstrated substantial inconsistency, with both under- and overestimation occurring across a broad range of GFR values (Pierrat et al., 2003; Björk et al., 2021; Filler et al., 2005). These limitations largely arise from fundamental physiological differences between children and adults where GFR does not reach adult levels until approximately the second year of life, and both muscle mass and creatinine production undergo rapid changes throughout childhood (Mian and Schwartz, 2017; Filler et al., 2014; Muhari-Stark and Burckart, 2018). The Modified Schwartz equation is most commonly used in paediatric practice and is often referenced in paediatric drug dosing tools (Schwartz et al., 2009). Although this equation has been found to improve GFR estimation in children, studies of children with and without kidney disease have reported similar trends in under- and overestimation of kidney function, risking inappropriate drug dosing (Pottel et al., 2016; Salvador et al., 2019; Blufpand et al., 2013). More recent creatinine-based equations such as the Chronic Kidney Disease in Children Under 25 years (CKiD-U25) and full age spectrum (FAS) equation, have demonstrated improved accuracy compared to the Modified Schwartz equation; however, they have only been validated in children over 1 and 2 years of age, respectively (Pottel et al., 2016; Pierce et al., 2021). Several other creatinine-based equations are available, but most have not been validated for clinical use (Mian and Schwartz, 2017; Muhari-Stark and Burckart, 2018).

This study aimed to develop a novel GFR equation for children, the Growth Adjusted Renal Output Wizard (GROW-GFR), using a population pharmacokinetic (popPK) model of tobramycin, an aminoglycoside antibiotic almost exclusively cleared by glomerular filtration, and (i) evaluate its performance against NM-GFR scans and (ii) compare its accuracy to other commonly used paediatric GFR equations.

## 2 Materials and methods

### 2.1 Creating the GROW-GFR equation

GFR was predicted using the estimated tobramycin clearance obtained from a published popPK model (Harwood et al., 2025). This tobramycin model was developed using data collected from a multicentre prospective study over a 2-year period (2020–2022) at the Royal Children’s Hospital (RCH), Melbourne and The Children’s Hospital at Westmead, Sydney, Australia (Ethics approval RCH HREC6562) (Harwood et al., 2025). The study included 63 children with a median age of 13.3 years (range 0.2–19.3) with cystic fibrosis (CF) who received intravenous (IV) tobramycin and had a median of 4 (range 1–34) tobramycin concentrations determined over the study period. In the present study, the published popPK model was not refitted. Instead, individual estimates of tobramycin clearance derived from the model were used as a surrogate measure of GFR. This assumption is supported by the fact that tobramycin is predominantly cleared unchanged by the kidneys, with approximately 90%–95% cleared by glomerular filtration (Tobramycin. Lexi, 2023).

### 2.2 Retrospective audit of nuclear medicine GFR scans

A retrospective audit of NM-GFR scans from children aged 0–19 years who presented to RCH, Melbourne was conducted over seven years (July 2016 to November 2023). Data were electronically extracted from the hospital’s electronic medical record system (EPIC®) by an experienced data analyst. These data included patient demographics (age, height, weight), NM-GFR result, serum creatinine levels, and comorbidities. Results were included if the NM-GFR was ≤150 mL/min; height had been measured within 1 month of the scan for infants aged <6 months, and within 3 months for those ≥6 months old; weight was recorded within 7 and 30 days of the scan for those aged <2 years and ≥2 years old, respectively; and serum creatinine was measured within 14 days of the scan, irrespective of age. Values for height, weight, and serum creatinine closest to the date of the NM-GFR result were used. Ethics approval was obtained from the RCH Ethics Committee (QA/104011/RCHM-2023).

At RCH, NM-GFR was measured using two methods during the study period. Both methods involved the injection of diethylenetriaminepentaacetic acid (^99m^Tc-DTPA) and the collection of two blood samples, one at 90 min and another at 180 min post-injection. Prior to October 2021, a weight-based dosing of ^99m^Tc-DTPA was used where plasma samples were analysed using a gamma counter. The mean counts from each sample were used to calculate GFR based on both the plasma sample counts and a reference sample. Results were reported in both mL/min and mL/min/1.73 m^2^. In October 2021, ^99m^Tc-DTPA dosing was transitioned to an activity-based approach and the methodology was conducted in accordance with the Fleming guidelines (Fleming et al., 2004). Plasma concentrations were derived as a percentage of the injected dose per litre and the slope-intercept method was used to calculate the GFR.

### 2.3 Performance of the GROW-GFR equation and four other paediatric GFR equations

For analysis, patients were stratified into four age groups (<3 months, 3 months to <2 years, 2–12 years, and >12 years old). The performance of the GROW-GFR equation and other paediatric equations ‒ Modified Schwartz equation (Schwartz et al., 2009), CKiD-U25 equation (Pierce et al., 2021), FAS equation, FAS-height (HT) equation (Pottel et al., 2016) – were compared to the NM-GFR results within each age group. As the other equations standardise eGFR to a body surface area (BSA) of 1.73 m^2^, this standardisation was removed using Equation 1 below where BSA was calculated using the Haycock equation (Equation 1) (Haycock et al., 1978).

$$\text{eGFR }\left(\text{mL}/\min\right)=\frac{\text{eGFR}\;\left(\text{mL}/\min/1.73m^{2}\right)\times \text{BSA}}{1.73}$$ (1)

The performance of each equation was assessed for all NM-GFR results and a subset of patients with an NM-GFR of ≤120 mL/min as accurate measurement of GFR above this threshold is rarely clinically indicated (Heilbron et al., 1991; Pottel et al., 2015; Jančič et al., 2022). The relationship between NM-GFR and the eGFR values calculated using GROW-GFR, Modified Schwartz, CKiD-U25, and FAS equations was assessed using linear regression and Bland-Altman plots. The percentage of estimates within 30% and 10% of the NM-GFR result, i.e., p30 and p10 accuracy, respectively, and the root mean square error (RMSE), measuring the average difference between estimated and measured GFR values, were determined. Bias was assessed as the median difference between eGFR and NM-GFR. All analyses were performed using the R language, through R studio version 4.4.1.

Generalised linear mixed-effects models were fitted to the accuracy (p30 and p10) of each equation using the glmer function from the lme4 package in R, with a binomial error family and a logit link function. A linear mixed-effects model was fitted to bias using the lmer function from the same package. All models (p30, p10 and bias) included GFR estimation method as a fixed effect, and random intercepts for measurement occasions nested within individuals to account for repeated measures. Model diagnostics of each model were assessed using residual plots and the check_model function to evaluate model fit and assumptions (Supplementary Table 1).

Estimated marginal means of accuracy (p30 and p10) and bias for each method were derived from the fitted model using the emmeans function from the emmeans package. The other estimation methods were contrasted with the GROW-GFR equation using the contrast command. Accuracy contrasts were reported in terms of their odds ratio, while bias was reported in terms of differences. A p value of <0.05 was used to determine statistical significance.

## 3 Results

### 3.1 Final GROW-GFR equation

The GROW-GFR equation is shown below (Equation 2), which considers the patient’s age (AGE; years), weight (WT; kg), height (HT; metres), and serum creatinine (CR; mg/dL).

$${\text{eGFR}}_{\text{GROW}‐\text{GFR}}\left(\text{mL}/\min\right)=\frac{1000}{60}\;\cdot \left[6.17\cdot \frac{{\left(\left(\text{AGE}\cdot 52\right)+40\right)}^{3.4}}{47.7^{3.4}+{\left(\left(\text{AGE}\cdot 52\right)+40\right)}^{3.4}}\cdot \left({\left(\frac{\text{WT}}{70}\right)}^{0.663}\right)\cdot \left({\left(\frac{41.3\cdot \left(\text{HT}/\text{CR}\right)}{127.3}\right)}^{0.4}\right)]\right.$$ (2)

### 3.2 Comparison of the GROW-GFR equation to nuclear medicine GFR scans

Overall, 1673 NM-GFR results from 754 children were extracted, of which 1,560 results from 711 children were eligible for inclusion. The clinical characteristics of the included patients are summarised in Table 1. The median (range) NM-GFR was 55 (0.5–150) mL/min. Nearly all children (710/711, 99.9%) had comorbidities, with malignancy being the most common (515/711, 72%), followed by prior solid organ or haematopoietic stem cell transplant (279, 39%), cardiac disease (91, 13%), and kidney disease (70, 10%). Notably, none of these patients had CF. Approximately half (54%) of children had more than one NM-GFR result available. Of these 1,560 results, 10 (0.6%) were from children aged <3 months, 211 (13.5%) 3 months to <2 years old, 845 (54.1%) 2–12 years old, and 494 (31.6%) >12 years old. Twelve (5.4%) children aged <2 years were born premature with a median gestational age of 33 (31–36) weeks.

**TABLE 1:** Patient characteristics.

Characteristics	Total number of scans (n = 1,560)	<3 m (n = 10)	3 m – <2 years (n = 211)	2–12 years (n = 845)	>12 years (n = 494)
Age, median (range), y	8.4 (0.1–19)	0.15 (0.1–0.23)	1.27 (0.27–1.98)	6.85 (2.02–12)	15.5 (12–19)
Height, median (range), m	1.28 (0.51–1.96)	0.57 (0.55–0.66)	0.78 (0.51–0.91)	1.18 (0.71–1.73)	1.64 (1.3–1.96)
Weight, median (range), kg	25.8 (3.92–114)	5.32 (3.92–6.59)	9.90 (4.34–16.1)	21 (8.60–69)	57.6 (27.3–114)
BSA, median (range), m^2^	0.96 (0.25–2.47)	0.29 (0.25–0.34)	0.47 (0.24–0.64)	0.82 (0.45–1.73)	1.64 (1.02–2.47)
Serum creatinine, median (range)	​	​	​	​	​
mg/dL	0.40 (0.14-2.93)	0.21 (0.16–0.27)	0.21 (0.14–0.67)	0.36 (0.14–1.27)	0.61 (0.26–2.93)
μmol/L	35 (12-259)	8.5 (14–24)	19 (12–59)	32 (12–112)	54 (23–259)
NM-GFR, median (range), mL/min	55 (0.5–150)	11 (5.6–19.5)	27 (9–86)	50 (9–148)	82 (0.5–150)

Characteristics	Total number of patients (n = 711)	<3 m (n = 10)	3 m – <2 years (n = 100)	2–12 years (n = 381)	>12 years (n = 220)
Male, n (%)	399 (56.1)	6 (60)	58 (58)	219 (57.5)	116 (52.7)
Comorbidities, n (%)	710 (99.9)	10 (100)	100 (100)	381 (100)	219 (99.5)
>1 comorbidity, n (%)	292 (38.3)	7 (70)	35 (35)	146 (38.3)	104 (47.2)
Type of comorbidity, n (%)					
Malignancy	515 (72.4)	2 (20)	76 (76)	284 (74.5)	153 (69.5)
Transplant	279 (39.2)	7 (70)	32 (32)	143 (37.5)	97 (44.1)
Cardiac	91 (12.8)	0 (0)	7 (7)	49 (12.9)	35 (15.9)
Kidney disease	70 (9.8)	0 (0)	6 (6)	34 (8.9)	30 (13.6)
Neurological	44 (6.2)	1 (10)	10 (10)	19 (5)	14 (6.4)
Endocrine	44 (6.2)	1 (10)	7 (7)	15 (3.9)	21 (9.5)
Haematological	25 (2.8)	0 (0)	0 (0)	17 (3.8)	8 (3.6)
Lung	22 (3.1)	0 (0)	2 (2)	11 (4.5)	9 (4.1)
Immunological	22 (3.1)	6 (60)	7 (7)	6 (1.6)	3 (1.4)
Other	6 (0.8)	0 (0)	0 (0)	3 (0.8)	3 (1.4)

BSA, body surface area (calculated using Haycock formula).

The accuracy and bias of the GROW-GFR equation in all children and those with an NM-GFR of ≤120 mL/min are outlined in Table 2. For all NM-GFR results, the overall p30 accuracy of the GROW-GFR equation was 76%. Notably, this was consistent across all age groups (80%, 74%, 75% and 78% in children aged <3 months, 3 months - <2 years, 2–12 years and >12 years, respectively). In children aged <3 months, and 3 months - <2 years, the RMSE was lower than in those aged 2–12 and >12 years (4.3% and 9.5% compared to 16% and 21%, respectively). An overall bias of −1.9 mL/min was observed across all ages. When limited to those with an NM-GFR ≤120 mL/min, the overall accuracy improved to 77%, with an overall bias of −0.2 mL/min.

**TABLE 2:** Performance of different GFR equations.

Data	GFR equation	P30 (%) (95% CI)	P10 (%) (95% CI)	RMSE (%) (95% CI)	Bias (mL/min) (95% CI)
All ages					
All NM-GFR (n = 1,560) NM-GFR ≤120 mL/min (n = 1,483)	GROW-GFR Schwartz CKiD-U25 FAS FAS-HT GROW-GFR Schwartz CKiD-U25 FAS FAS-HT	76.1 (73.9, 78.1) 46.4 (43.9, 48.8) - - - 77.1 (74.9, 79.2) 44.3 (41.8, 46.8) - - -	30.3 (28, 32.6) 18.3 (16.4, 20.3) - - - 31.5 (29.2, 33.9) 17.2 (15.3, 19.2) - - -	17.1 (16.5, 17.7) 27.7 (26.8, 28.7) - - - 14.9 (14.4, 15.5) 27.6 (26.7, 28.7) - - -	−1.9 (−2.8, −1.1) 17.3 (16.2, 18.4) - - - −0.2 (−0.9, 0.6) 18.2 (17.2, 19.3) - - -
< 3 months					
All NM-GFR (n = 10) NM-GFR ≤120 mL/min (n = 10)	GROW-GFR Schwartz CKiD-U25 FAS FAS-HT GROW-GFR Schwartz CKiD-U25 FAS FAS-HT	80 (49, 94.3) 10 (1.8, 40.4) - - - 80 (49, 94.3) 10 (1.8, 40.4) - - -	30 (10.8, 60.3) 0 (0, 27.8) - - - 30 (10.8, 60.3) 0 (0, 27.8) - - -	4.3 (3.0, 7.9) 9.3 (6.4, 17) - - - 4.3 (3.0, 7.9) 9.3 (6.4, 17) - - -	−2.6 (−4.9, −0.4) 8 (4.9, 11.1) - - - −2.6 (−4.9, −0.4) 8 (4.9, 11.1) - - -
3 months - < 2 years					
All NM-GFR (n = 211) NM-GFR ≤120 mL/min (n = 211)	GROW-GFR Schwartz CKiD-U25* FAS FAS-HT GROW-GFR Schwartz CKiD-U25* FAS FAS-HT	74.4 (68.1, 79.8) 34.6 (28.5, 41.2) 60.1 (51.8, 67.9) - - 74.4 (68.1, 79.8) 34.6 (28.5, 41.2) 60.1 (51.8, 67.9 - -	26.5 (21, 32.9) 13.3 (9.3, 18.5) 20.3 (14.4, 27.8) - - 26.5 (21, 32.9) 13.3 (9.3, 18.5) 20.3 (14.4, 27.8) - -	9.5 (8.7, 10.5) 16.4 (15, 18.1) 11.6 (10.4, 13.2) - - 9.5 (8.7, 10.5) 16.4 (15, 18.1) 11.6 (10.4, 13.2) - -	−1.9 (−3.2, −0.6) 11 (9.4, 12.7) 4.1 (2.3, 6) - - −1.9 (−3.2, −0.6) 11 (9.4, 12.7) 4.1 (2.3, 6) - -
2–12 years					
All NM-GFR (n = 845) NM-GFR ≤120 mL/min (n = 830)	GROW-GFR Schwartz CKiD-U25 FAS FAS-HT GROW-GFR Schwartz CKiD-U25 FAS FAS-HT	75.3 (72.3, 78.1) 43.8 (40.5, 47.2) 60.7 (57.4, 64) 52.9 (49.5, 56.2) 55.9 (52.5, 59.2) 76 (73, 78.8) 43 (39.7, 46.4) 60.4 (57, 63.6) 52.3 (48.9, 55.7) 55.4 (52, 58.8)	31 (28, 34.2) 16.7 (14.3, 19.4) 23.3 (20.6, 26.3) 21.2 (18.6, 24.1) 23.2 (20.5, 26.2) 31.6 (28.5, 34.8) 16.1 (13.8, 18.8) 23.3 (20.5, 26.3) 21 (18.3, 23.9) 22.9 (20.2, 25.9)	16 (15.3, 16.8) 26 (24.8, 27.3) 20.4 (19.4, 21.4) 22.8 (21.8, 23.9) 22 (21, 23.1) 14.7 (14, 15.5) 26 (24.8, 27.3) 20.2 (19.3, 21.2) 22.8 (21.7, 23.9) 21.9 (20.9, 23)	−3.2 (−4.2, −2.1) 17.2 (15.9, 18.5) 8.9 (7.6, 10.1) 12.4 (11.1, 13.7) 11.2 (9.9, 12.5) −2.4 (−3.4, −1.4) 17.6 (16.3, 18.9) 9.3 (8.1, 10.6) 12.8 (11.6, 14.1) 11.6 (10.3, 12.9)
> 12 years					
All NM-GFR (n = 494) NM-GFR ≤120 mL/min (n = 435)	GROW-GFR Schwartz CKiD-U25 FAS FAS-HT GROW-GFR Schwartz CKiD-U25 FAS FAS-HT	78.1 (74.3, 81.6) 56.5 (52.1, 60.8) 53.6 (49.2, 58) 46.4 (42, 50.8) 48.6 (44.2, 53) 80.5 (76.5, 83.9) 52.2 (47.5, 56.8) 49.2 (44.5, 53.9) 41.4 (36.9, 46.1) 43.7 (39.1, 48.4)	30.6 (26.7, 34.8) 23.5 (20, 27.4) 21.1 (17.7, 24.9) 16.6 (13.6, 20.1) 18.6 (15.4, 22.3) 33.8 (29.5, 38.4) 21.4 (17.8, 25.5) 19.3 (15.9, 23.3) 13.6 (10.7, 17.1) 16.1 (12.9, 19.8)	21 (19.7, 22.4) 33.9 (31.9, 36.2) 35.3 (33.2, 37.7) 39.6 (37.2, 42.2) 39.7 (37.4, 42.4) 17.3 (16.3, 18.6) 34.4 (32.2, 36.8) 35.8 (33.5, 38.3) 40.5 (38, 43.4) 40.2 (37.7, 43)	0.2 (−1.7, 2) 20.3 (17.9, 22.7) 22 (19.6, 24.5) 27.8 (25.3, 30.3) 27.2 (24.6, 29.7) 4.9 (3.3, 6.5) 23.1 (20.7, 25.5) 24.6 (22.2, 27) 30.6 (28, 33.1) 29.5 (26.9, 32)

NM-GFR, nuclear medicine glomerular filtration rate; GFR, glomerular filtration rate; P30, percentage of estimates within 30%; P10, percentage of estimates within 10%; RMSE, residual mean standard error.

* Children ≥1 year old.

### 3.3 Comparison of the GROW-GFR to other paediatric GFR equations

Figure 1 shows the performance of the GROW-GFR, Modified Schwartz, CKiD-U25, FAS, and FAS-HT equations in comparison to NM-GFR. Across all age groups, the GROW-GFR equation demonstrated substantially higher p30 accuracy (76%) compared to the Modified Schwartz (46%). In children aged ≥2 years old, GROW-GFR maintained higher accuracy (76%), compared to the Modified Schwartz (49%), CKiD-U25 (58%), FAS (51%), and FAS-HT (53%) equations. This was also reflected in the RMSE comparison, with the GROW-GFR equation yielding a considerably lower RMSE (18%) compared to the Modified Schwartz (29%), CKiD-U25 (27%) and the FAS and FAS-HT equations (30%) in the ≥2 years old group. A similar trend was observed for p10 accuracy (Table 2). Furthermore, the p30 and p10 accuracy of the GROW-GFR equation was consistent across all age groups, including children <2 years old, which cannot be predicted by the FAS and FAS-HT equations. In the latter age group, GROW-GFR achieved a p30 accuracy of 80% in children <3 months and 74% in those aged 3 months - <2 years, significantly outperforming the Modified Schwartz equation, which had p30 accuracies of only 10% and 35%, respectively. GROW-GFR also outperformed the CKiD-U25 equation in children between 1–2 years old (75% vs. 40%, respectively).

**FIGURE 1:**
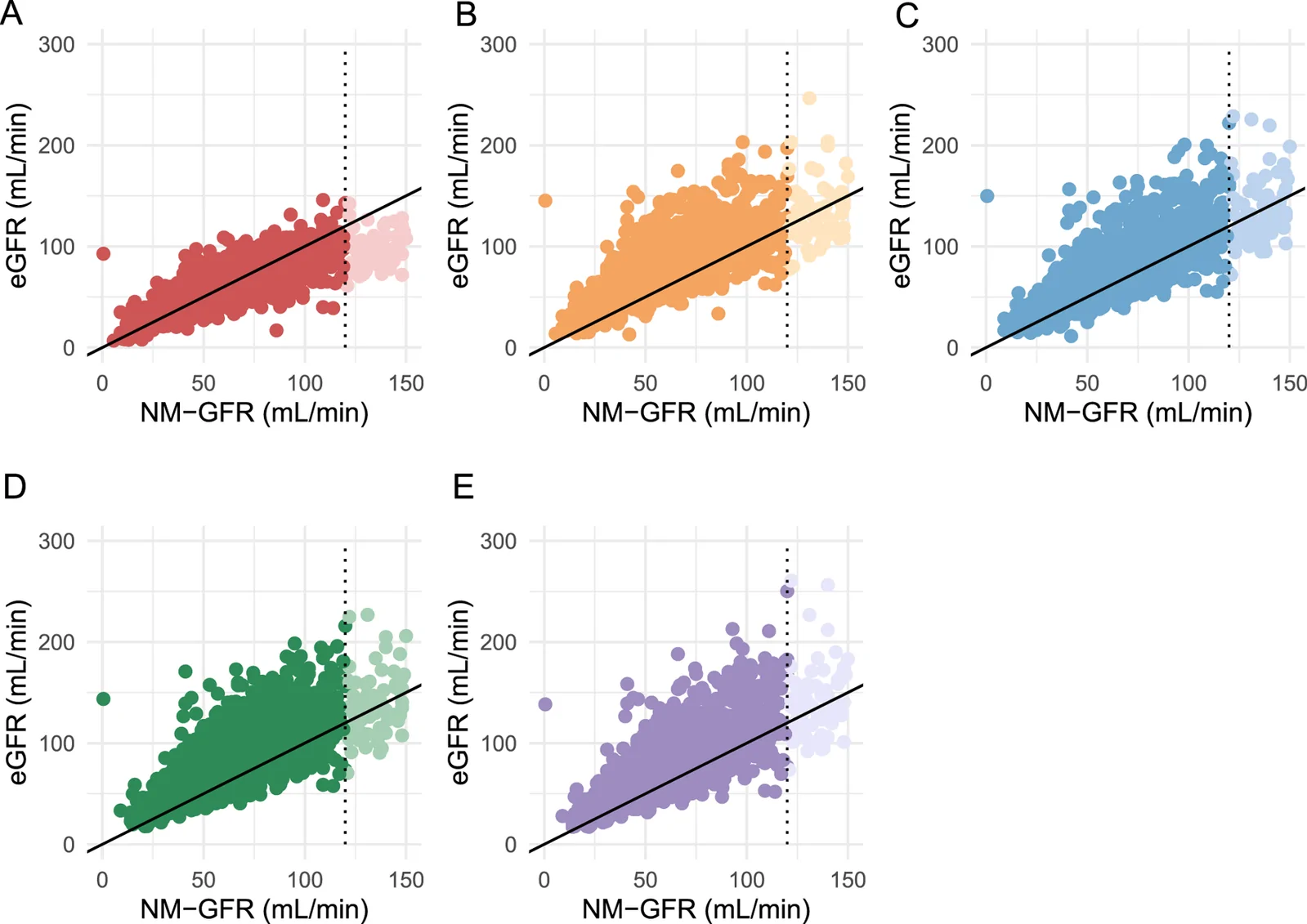
Relationship between estimated glomerular filtration rate (eGFR) calculated using the GROW-GFR (**(A)**, red), Modified Schwartz (**(B)**, orange), CKiD-U25 (**(C)**, blue), FAS (**(D)**, green), and FAS-HT (**(E)**, purple) equations and GFR measured by nuclear medicine scans (NM-GFR) in all children. All GFR values are expressed in mL/min. The solid horizontal line is the line of identity, the dotted vertical line indicates an NM-GFR of 120 mL/min. Lighter-shaded data points represent NM-GFR values exceeding 120 mL/min.

In children of all ages, the GROW-GFR equation demonstrated statistically significantly lower overall bias (−1.9 mL/min) compared to the Modified Schwartz (17.3 mL/min) (Figure 2; Table 2). This was also seen in children aged ≥2 years where GROW-GFR again showed substantially lower bias (−1.9 mL/min) vs. the Modified Schwartz (18.4 mL/min), CKiD-U25 (13.7 mL/min), FAS (18 mL/min), and FAS-HT (17.1 mL/min) equations. When stratified by age, the Modified Schwartz equation overestimated GFR, with a mean bias of 10.9 mL/min in children <2 years, whereas the GROW-GFR equation slightly underestimated GFR in this group, with the mean bias remaining consistent at −1.9 mL/min. Similarly, in children >12 years, the other equations exhibited greater bias, overestimating GFR by 20.3 mL/min (Modified Schwartz), 22 mL/min (CKiD-U25), 27.8 mL/min (FAS), and 27.2 mL/min (FAS-HT), while the GROW-GFR equation showed minimal bias of 0.2 mL/min.

**FIGURE 2:**
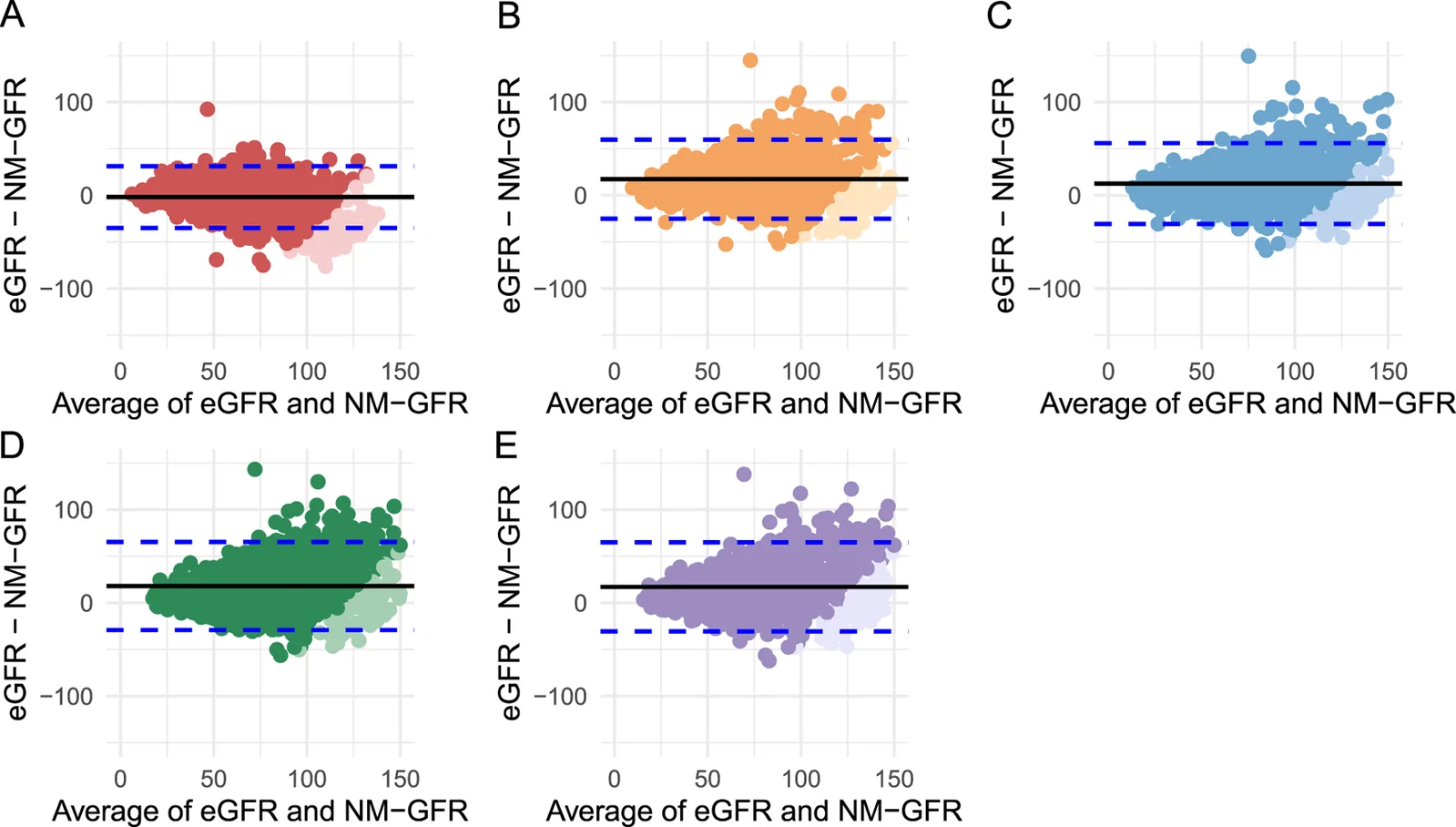
Bland-Altman plots for GROW-GFR (**(A)**, red), Modified Schwartz (**(B)**, orange), CKiD-U25 (**(C)**, blue), FAS (**(D)**, green), and FAS-HT (**(E)**, purple) equations versus nuclear medicine GFR (NM-GFR) in all children. Horizontal solid line represents the mean difference between eGFR and NM-GFR, upper and lower horizontal dashed blue lines represent ±1.96 SD. Lighter-shaded data points represent NM-GFR values exceeding 120 mL/min.

After fitting estimates to a generalised linear mixed-effects model, the overall p30 accuracy increased to 80% (95% CI 78–83) for all NM-GFR results and remained high across all age groups (80%, 77%, 94% and 96% in children aged <3 months, 3 months - <2 years, 2–12 years and >12 years, respectively) (Supplementary Table 2). The mean bias across all ages also remained similar at −2.5 mL/min (95% CI -3.7–1.3) with less bias in those aged >12 years (0.1 mL/min 95% CI -3.0–3.1 mL/min). Pairwise contrasts of the p30 accuracy of the other GFR equations in comparison to the GROW-GFR showed the odds ratio for p30 accuracy was 0.21 across all age groups (Modified Schwartz vs. GROW-GFR, 95% CI 0.18–0.25, p < 0.001) (Supplementary Table 3). For children ≥2 years, the CKiD-U25, FAS-HT and FAS had an odds ratio of 0.16 (95% CI 0.12–0.22, p < 0.001), 0.08 (95% CI 0.06–0.11, p < 0.001) and 0.10 (95% CI 0.07–0.14, p < 0.001), respectively.

## 4 Discussion

Our GROW-GFR equation, utilising a popPK model of tobramycin (a 90%–95% renally cleared drug) (Harwood et al., 2025), showed greater accuracy in estimating NM-GFR results compared to existing paediatric-specific creatinine-based GFR equations, with higher p30 accuracy (76% vs. 49%–58%) in children ≥2 years old. Importantly, this p30 accuracy remained high in children <1 and <2 years of age, where other equations either fail to predict (CKiD-U25, FAS, FAS-HT) or perform poorly (Modified Schwartz). After fitting estimates to a generalised linear mixed-effects model, the p30 accuracy of GROW-GFR was 80% overall, and 77% and 94% in children <2 and ≥2 years old, respectively. Notably, our equation incorporates a published renal maturation model that accounts for changes in GFR with increasing post-menstrual age (Anderson and Holford, 2009), which may contribute to its improved performance in younger age groups compared to equations which do not account for this. Given the critical role of GFR estimation in clinical decision-making, over- or underestimating GFR can have serious consequences. In oncology patients requiring chemotherapy, overestimating GFR may lead to overdosing, and inappropriate selection of chemotherapy agents, increasing the risk of adverse effects, including kidney injury. Underestimating GFR can result in underdosing and exclusion of effective agents, compromising treatment efficacy (Casal et al., 2019; Jia et al., 2015). Similar consequences apply to dosing antimicrobial agents which are cleared by the kidneys (Clifford et al., 2022).

Despite the Modified Schwartz equation being the most frequently used in clinical practice, its accuracy and bias have been questioned. A study of 96 children aged 0.25 to 17.5 years showed a p30 accuracy of 53% and median bias of 15.5 mL/min/1.73 m^2^, similar to that shown in this study (46% and 17.3 mL/min, respectively) (Salvador et al., 2019). Variability in accuracy and bias between younger and older children has also been reported with a p30 accuracy of 11% and bias of 32 mL/min/1.73 m^2^ in children <2 years compared to 84% and 2.6 mL/min/1.73 m^2^ in those aged ≥13 years (Blufpand et al., 2013). While the FAS equations performed similarly to the Modified Schwartz equation in our study, a previous study of 735 children showed the FAS equations to have significantly lower bias compared to the Modified Schwartz equation (−1.7, −2.7 for the FAS and FAS-HT compared to 6.0 mL/min/1.73 m^2^ Modified Schwartz); however, it's inability to be used in children <2 years of age remains a key limitation (Pottel et al., 2016). The CKiD-U25 equation has also been shown to outperform the Modified Schwartz equation in 221 children, with higher p30 accuracy (73% vs. 65%) and lower bias (3.0 vs. 12.8 mL/min/1.73 m^2^); however, like the FAS equations, it is not applicable to young children <1 year of age (Kim et al., 2026). The superior performance of the GROW-GFR equation shown in this study compared to the Modified Schwartz, CKiD-U25, and FAS equations may be attributed not only to the inclusion of the maturation function, but also potentially to the lesser influence of serum creatinine (exponent 0.4) in this equation compared with other creatinine-based equations. Our equation also shows comparable performance to commonly used adult GFR equations. A study in 117 healthy adults compared adult GFR equations, including Cockroft-Gault and MDRD, with NM-GFR measured using ^99m^Tc-DTPA and reported p30 accuracies of 58% and 69%, respectively (Lin et al., 2003). The authors noted that the Cockroft-Gault equation overestimated GFR values (bias 16.8 mL/min/1.73 m^2^), while MDRD underestimated these values (−15.2 mL/min/1.73 m^2^). This underestimation was also noted in our equation, albeit to a lesser extent, with a bias of −1.9 ml/min.

Although the gold standard for GFR measurement is a 24-h urinary clearance of exogenous markers such as iohexol, ^51^Cr-EDTA, and ^99m^Tc-DTPA, these are rarely done in paediatric practice as they require a continuous IV infusion, repetitive blood and urine collections, and often bladder catheterisation (Stevens et al., 2006; Pierrat et al., 2003; Fleming et al., 2004; Jančič et al., 2022; Pottel, 2017). Consequently, NM-GFR scans are often used as a surrogate as they require fewer blood samples; however, these scans remain time-consuming, expensive and invasive due to the injection of exogenous and often radioactive markers. As a result, current guidelines recommend reserving gold standard measurements such as NM-GFR scans for specific clinical circumstances, such as aiding clinical decision-making for patients with malnutrition, heart failure, or those receiving broad-spectrum antibiotics that reduce extrarenal elimination (Kidney Disease: Improving Global Outcomes CKD Work Group, 2024). For routine GFR assessment, creatinine-based or combined creatinine-cystatin C equations are recommended, with the latter preferred when a more accurate estimate is needed to guide clinical decisions, such as diagnosing and staging chronic kidney disease or adjusting drug dosages (Stevens et al., 2006; Rahn et al., 1999; Kidney Disease: Improving Global Outcomes CKD Work Group, 2024; Mathias et al., 2000). Therefore, cystatin C has gained preference as an alternative marker as its production remains unaffected by sex, age, and muscle mass (Vinge et al., 1999; Dharnidharka et al., 2002; Roos et al., 2007; Bouvet et al., 2006; Bökenkamp et al., 1998; Finney et al., 2000). However, this assay is less accessible and is associated with higher costs compared to serum creatinine, thus is not routinely assessed in clinical practice (Tummalapalli et al., 2020). While numerous comparative studies have demonstrated that cystatin C-based equations are more accurate and less biased than creatinine-based equations (Skidmore et al., 2024; Zappitelli et al., 2006; Poventud-Fuentes et al., 2022; Filler and Lepage, 2003), the GROW-GFR equation appears to perform just as well, if not better, than the cystatin C-based GFR equations used in children with a p30 of 80% and bias of −2.5 ml/min (92% and −2.4 ml/min in children ≥2 years). This accuracy is similar to the Filler and Lepage cystatin C-equation (Filler and Lepage, 2003) (p30 85%, bias −2.7 mL/min/1.73 m^2^), and notably better than the CysEq (Zappitelli et al., 2006), Bokenkamp (Bökenkamp et al., 1998), and Grubb equations (Grubb et al., 2005), which showed either lower accuracy (p30 81%, 56%, and 50%) and/or higher bias (15.8, −30.8, and −38.7 mL/min/1.73 m^2^).

BSA standardisation is conventionally applied in adults to facilitate comparison between individuals and to define normal reference values. However, the reference value of 1.73 m^2^ reflects the mean BSA of young American adults in 1925 (Chew-Harris et al., 2015). This value is outdated and inappropriate for individuals whose BSA differs substantially, such as children, or those with conditions associated with muscle wasting, or obesity (Heaf, 2007; Redal-Baigorri et al., 2014; Delanaye et al., 2009; Peters et al., 2000). Multiple studies comparing standardised and absolute GFR across such populations have consistently shown greater discrepancies in individuals with a BSA of <1.60 or >2.00 m^2^, the former representing most of the paediatric population (Redal-Baigorri et al., 2014; Redal-Baigorri et al., 2013; El Edelbi et al., 2012; Brandt et al., 2006; Correia-Costa et al., 2016). A study of 895 adult oncology patients showed that GFR was overestimated by 10.08 mL/min (11.46%) in individuals with a BSA <1.60 m^2^ and underestimated by up to −20.76 mL/min (−23.59%) when the BSA exceeded 2.00 m^2^ (Redal-Baigorri et al., 2014). Similar concerns have been raised in paediatric populations, where studies have shown that indexing BSA to the adult reference BSA may lead to misrepresentation of kidney function in infants and children, particularly for those with atypical body composition, including obesity (Brandt et al., 2006; Correia-Costa et al., 2016). This aligns with several kidney and drug dosing guidelines, including KDIGO, which recommend that drug dosing in children and adults with extreme body sizes should be based on absolute rather than standardised GFR (Kidney Disease: Improving Global Outcomes CKD Work Group, 2024; European Medicines Agency, 2016; U.S. Food and Drug Administration, 2024). In adults, the CKD-EPI equation conventionally reported the GFR standardised to BSA, however, unstandardised CKD-EPI values have been shown to provide greater accuracy for drug dosing and for predicting aminoglycoside clearance (Chew-Harris et al., 2015; Chin et al., 2013; Pai et al., 2011). Although several alternative paediatric eGFR equations estimating absolute GFR have being developed (Heilbron et al., 1991; Bouvet et al., 2006; Léger et al., 2002; Brandt et al., 2003), these have not yet been incorporated into clinical guidelines, which continue to recommend BSA-adjusted equations such as the Modified Schwartz, CKiD-U25, and FAS equations.

This study had some limitations. Firstly, the equation was originally developed from a relatively small cohort of 63 children with CF, which may raise concerns regarding its generalisability to non-CF populations, particularly given the lack of consensus on whether CF affects tobramycin clearance (David, 1990; de Groot and Smith, 1987; Levy et al., 1984; Hennig et al., 2013). However, in our study, which included a large and diverse paediatric cohort of 711 children (1,560 observations) without CF, the equation demonstrated good performance, suggesting that any CF-specific effects have minimal impact on its validity in broader paediatric populations. This population predominantly consisted of children with normal renal function, therefore, the performance of the GROW-GFR equation in populations with significantly reduced kidney function warrants further investigation. We compared the performance of our equation with several commonly used paediatric eGFR equations. NM-GFR values were capped at ≤150 mL/min as GFR measurement is most often required for patients with renal impairment; thus, the equation may not accurately predict renal hyperfiltration (GFR >150 mL/min). GFR was measured using NM-GFR scans with ^99m^Tc-DTPA injection (Fleming et al., 2004; Blaufox et al., 1996). Although, studies have shown comparable accuracy with the gold-standard inulin method (Barbour et al., 1976; Rehling et al., 1984), some have identified that ^99m^Tc-DTPA overestimates at low and underestimates at high GFRs (De Santo et al., 1999). In addition, ^99m^Tc-DTPA exhibits 10%–13% protein binding which may contribute to slight underestimation of GFR; however, this is comparable to other established GFR markers (Rehling et al., 2001). A similar negative bias was observed in our plots, therefore, the use of ^99m^Tc-DTPA may partially account for the bias identified with our equation. Hydration status at the time of NM-GFR measurement is not routinely recorded and therefore could not be evaluated in this study. As hydration status may influence renal functional reserve and measured GFR, this may be a potential source of variability in the NM-GFR results. A change in NM-GFR methodology during the study period may have introduced variability in the analysis. Sensitivity analyses were conducted to assess the variability by comparing the performance of each GFR equation before and after the transition. This demonstrated similar results across both periods, suggesting that the change in methodology had minimal impact on overall findings (Supplementary Table 5). Due to the observational nature of the study, height, weight and serum creatinine measurements were not always recorded on the day of the scan which may affect the accuracy of the GFR equations. To address this, age-specific time windows for prior or subsequent measurements were applied to ensure a reasonable approximation of height and weight at the time of the scan, with a general window applied for serum creatinine for all patients. Finally, whilst creatinine is the most widely used endogenous marker, its reliability in children is limited due to strong correlation with muscle mass, which varies significantly throughout childhood (Mian and Schwartz, 2017; Filler et al., 2014; Vinge et al., 1999). Our equation mitigates this by incorporating height, weight, and age, improving adjustment for muscle mass variation.

Overall, our GROW-GFR equation, based on a popPK model of tobramycin, showed better accuracy and lower bias than other paediatric GFR equations. Its accuracy remained consistent across all age groups, including children <2 years, outperforming the Modified Schwartz equation. This equation should replace existing tools used for estimating GFR in children to guide the dosing of renally excreted drugs such as chemotherapeutics and antibiotics as it provides a more accurate estimation of absolute GFR, aligning with current guideline recommendations.

## Data Availability

The raw data supporting the conclusions of this article will be made available by the authors upon reasonable request.
