# Supplementary material for: Refining absolute GFR estimation in children: development and validation of the GROW-GFR equation
Source: Front Pharmacol. 2026 Oct 22;17:1855031. doi: 10.3389/fphar.2026.1855031 (PMC13736570; doi:10.3389/fphar.2026.1855031)
Supplement: Supplementary file 1 [file Supplementaryfile1.docx]

**The GROW-GFR Calculator: A Novel Tool for Estimating Glomerular Filtration Rate in Children – Supplementary Materials**

**Contents:**

**eEquation 1 –** Haycock Equation

**Supplementary Table 1** – Posterior predictive check of (generalised) linear mixed effects models

**Supplementary Table 2 –** Performance of different eGFR equations when fit to (generalised) linear models

**Supplementary Table 3 –** Comparative Performance of GROW-GFR equation against other paediatric eGFR equations when fit to (generalised) linear models

**Supplementary Table 4**– Performance of different eGFR equations from NM-GFR results available before or from October 2021

**eEquation 1** **– Haycock Equation**

$$BSA=0.024265 \times{Height (cm)}^{0.3964}\times{Weight (kg)}^{0.5378}$$

**Supplementary Table 1 – Posterior predictive check of (generalised) linear mixed effects models**

| Data | P30 | P10 | Bias |
| --- | --- | --- | --- |
| All ages | | | |
| All NM-GFR |  |  |  |
| NM-GFR ≤ 120 ml/min |  |  |  |
| < 2 years | | | |
| All NM-GFR |  |  |  |
|  |  |  |  |
| < 2 years | | | |
| NM-GFR ≤ 120 ml/min |  |  |  |
| < 3 months | | | |
| All NM-GFR |  |  |  |
| NM-GFR ≤ 120 ml/min |  |  |  |
|  |  |  |  |
| 3 months - < 2 years | | | |
| All NM-GFR |  |  |  |
| NM-GFR ≤ 120 ml/min |  |  |  |
| 1 - < 2 years |  |  |  |
| All NM-GFR |  |  |  |
| NM-GFR ≤ 120 ml/min |  |  |  |
| ≥ 2 years | | | |
| All NM-GFR |  |  |  |
| NM-GFR ≤ 120 ml/min |  |  |  |
| 2 - 12 years |  |  |  |
| All NM-GFR |  |  |  |
| NM-GFR ≤ 120 ml/min |  |  |  |
| > 12 years | | | |
| All NM-GFR |  |  |  |
| NM-GFR ≤ 120 ml/min |  |  |  |

NM-GFR, nuclear medicine glomerular filtration rate; P30, percentage of estimates within 30%; P10, percentage of estimates within 10%

**Supplementary Table 2 – Performance of different eGFR equations when fit to (generalised) linear models**

| Data | eGFR equation | P30 (%) (95% CI) | P10 (%) (95% CI) | | Bias (ml/min) (95% CI) | |
| --- | --- | --- | --- | --- | --- | --- |
| All ages | | | | | | |
| All NM-GFR (n=1560)  NM-GFR ≤ 120 ml/min (n=1483) | GROW-GFR  Schwartz  CKiD-U25  FAS  FAS-HT  GROW-GFR  Schwartz  CKiD-U25  FAS  FAS-HT | 80 (78, 83)  47 (43, 50) -  -  -  82 (79, 84)  44 (41, 48) -  -  - | 30 (28, 32)  18 (16, 20)  -  -  -  31 (29, 34)  17 (15, 19)  -  -  - | | -2.50 (-3.73, -1.28)  16.7 (15.5, 18.0)  -  -  -  -0.54 (-1.72, 0.64)  17.9 (16.7, 19.0)  -  -  - | |
| < 2 years | | | | | |  |
| All NM-GFR (n=221)  NM-GFR ≤ 120 ml/min (n=221) | GROW-GFR  Schwartz  CKiD-U25  FAS  FAS-HT  GROW-GFR  Schwartz  CKiD-U25  FAS  FAS-HT | 77 (70, 83)  33 (26, 40) -  -  -  77 (70, 83)  33 (26, 40) -  -  - | 27 (21, 33)  13 (9, 18)  -  -  -  27 (21, 33)  13 (9, 18)  -  -  - | | -2.04 (-3.65, -0.43)  10.8 (9.17, 12.4)  -  -  -  -2.04 (-3.65, -0.43)  10.8 (9.17, 12.4)  -  -  - | |
| < 3 months | | | |  |  |  |
| All NM-GFR (n=10)  NM-GFR ≤ 120 ml/min (n=10) | GROW-GFR  Schwartz  CKiD-U25  FAS  FAS-HT  GROW-GFR  Schwartz  CKiD-U25  FAS  FAS-HT | 80 (46, 95)  10 (1, 47)  -  -  -  80 (46, 95)  10 (1, 47)  -  -  - | 30 (10, 62)  0 (0, 100)  -  -  -  30 (10, 62)  0 (0, 100)  -  -  - | | -2.63 (-5.66, 0.40)  8.00 (4.97, 11.0)  -  -  -  -2.63 (-5.66, 0.40)  8.00 (4.97, 11.0)  -  -  - | |
| 3 months - < 2 years | | | |  |  |  |
| All NM-GFR (n=211)  NM-GFR ≤ 120 ml/min (n=211) | GROW-GFR  Schwartz  CKiD-U25  FAS  FAS-HT  GROW-GFR  Schwartz  CKiD-U25  FAS  FAS-HT | 77 (70, 83)  34 (27, 42)  -  -  -  77 (70, 83)  34 (27, 42)  -  -  - | 27 (21, 33)  13 (9, 19)  -  -  -  27 (21, 33)  13 (9, 19)  -  -  - | | -1.98 (-3.67, -0.28)  10.9 (9.25, 12.6)  -  -  -  -1.98 (-3.67, -0.28)  10.9 (9.25, 12.6)  -  -  - | |
| 1 - < 2 years |  |  |  | |  | |
| All NM-GFR (n=211)  NM-GFR ≤ 120 ml/min (n=211) | GROW-GFR  Schwartz  CKiD-U25  FAS  FAS-HT  GROW-GFR  Schwartz  CKiD-U25  FAS  FAS-HT | 83 (71, 90)  35 (23, 50)  65 (50, 77)  -  -  83 (71, 90)  35 (23, 50)  65 (50, 77)  -  - | 27 (20, 35)  17 (12, 25)  20 (14, 28)  -  -  27 (20, 35)  17 (12, 25)  20 (14, 28)  -  - | | -2.46 (-4.81, -0.10)  11.1 (8,77, 13.5)  3.90 (1.55, 6.26)  -  -  -2.46 (-4.81, -0.10)  11.1 (8,77, 13.5)  3.90 (1.55, 6.26)  -  - | |
| ≥ 2 years | | | |  |  |  |
| All NM-GFR (n=1339)  NM-GFR ≤ 120 ml/min (n=1265) | GROW-GFR  Schwartz  CKiD-U25  FAS  FAS-HT  GROW-GFR  Schwartz  CKiD-U25  FAS  FAS-HT | 94 (92, 95)  51 (44, 57)  71 (65. 77)  55 (49, 62)  61 (55, 68)  95 (94, 97)  46 (39, 53)  70 (63, 75)  51 (44, 59)  58 (51, 65) | 24 (21, 27)  12 (10, 14)  15 (12, 17)  12 (10, 14)  14 (12, 16)  26 (22, 29)  10 (9, 13)  14 (12, 17)  11 (9, 13)  13 (11, 15) | | -2.35 (-3.93, -0.77)  17.9 (16.4, 19.5)  13.3 (11.7, 14.9)  17.6 (16.0, 19.2)  16.7 (15.1, 18.3)  -0.03 (-1.60, 1.54)  19.4 (17.8, 20.9)  14.4 (12.8, 16.0)  18.8 (17.2, 20.3)  17.6 (16.0, 19.2) | |
| 2 – 12 years | | | |  |  |  |
| All NM-GFR (n=845)  NM-GFR ≤ 120 ml/min (n=830) | GROW-GFR  Schwartz  CKiD-U25  FAS  FAS-HT  GROW-GFR  Schwartz  CKiD-U25  FAS  FAS-HT | 94 (91, 95)  42 (34, 50)  77 (70, 83)  62 (54, 70)  68 (60, 75)  94 (92, 96)  40 (32, 49)  77 (70, 83)  62 (53, 69)  68 (60, 75) | 25 (21, 29)  10 (8, 12)  16 (13, 19)  14 (11, 17)  16 (13, 19)  25 (21, 30)  9 (7, 12)  16 (13, 20)  14 (11, 17)  16 (13, 19) | | -3.86 (-5.41, -2.30)  16.5 (15.0, 18.1)  8.16 (6.61, 9.72)  11.7 (10.1, 13.2)  10.5 (8.9, 12.1)  -2.98 (-4.53, -1.43)  17.0 (15.5, 18.6)  8.71 (7.16, 10.3)  12.2 (10.7, 13.8)  11.0 (9.42, 12.5) | |
| > 12 years | | | |  |  |  |
| All NM-GFR (n=494)  NM-GFR ≤ 120 ml/min (n=435) | GROW-GFR  Schwartz  CKiD-U25  FAS  FAS-HT  GROW-GFR  Schwartz  CKiD-U25  FAS  FAS-HT | 96 (93, 97)  69 (58, 78)  63 (51, 73)  45 (33, 57)  50 (39, 62)  98 (96, 99)  62 (47, 75)  54 (39, 68)  31 (20, 45)  38 (25, 52) | 23 (18, 28)  15 (11, 19)  13 (9, 17)  9 (6, 12)  10 (8, 14)  26 (20, 33)  12 (9, 17)  10 (7, 14)  6 (4, 9)  8 (5, 11) | | 0.05 (-2.99, 3.09)  20.2 (17.1, 23.2)  21.9 (18.8, 24.9)  27.6 (24.6, 30.7)  27.1 (24, 30.1)  5.17 (2.17, 8.18)  23.4 (20.4, 26.4)  24.9 (21.9, 27.9)  30.8 (27.8, 33.8)  29.7 (26.7, 32.8) | |

NM-GFR, nuclear medicine glomerular filtration rate; eGFR, estimated glomerular filtration rate; P30, percentage of estimates within 30%; P10, percentage of estimates within 10%

**Supplementary Table 3 – Comparative Performance of GROW-GFR equation against other paediatric eGFR equations when fit to (generalised) linear models**

| Data | EGFR equation | P30 odds ratio (95% CI) | P10 odds ratio (95% CI) | | Bias (ml/min) (95% CI) | |
| --- | --- | --- | --- | --- | --- | --- |
| All ages | | | | | | |
| All NM-GFR (n=1560)  NM-GFR ≤ 120 ml/min (n=1483) | Schwartz  CKiD-U25  FAS  FAS-HT  Schwartz  CKiD-U25  FAS  FAS-HT | 0.21 (0.18, 0.25) *  -  -  -  0.17 (0.14 0.21) *  -  -  - | 0.51 (0.43, 0.61) *  -  -  -  0.45 (0.37, 0.53) *  -  -  - | | 19.2 (18.4, 20.1) *  -  -  -  18.4 (17.6, 19.2) *  -  -  - | |
| < 2 years | | | | | |  |
| All NM-GFR (n=221)  NM-GFR ≤ 120 ml/min (n=221) | Schwartz  CKiD-U25  FAS  FAS-HT  Schwartz  CKiD-U25  FAS  FAS-HT | 0.14 (0.09, 0.23) *  -  -  -  0.14 (0.09, 0.23) *  -  -  - | 0.40 (0.24, 0.65) *  -  -  -  0.40 (0.24, 0.65) *  -  -  - | | 12.8 (11.7, 13.9) *  -  -  -  12.8 (11.7, 13.9) *  -  -  - | |
| < 3 months | | | |  |  |  |
| All NM-GFR (n=10)  NM-GFR ≤ 120 ml/min (n=10) | Schwartz  CKiD-U25  FAS  FAS-HT  Schwartz  CKiD-U25  FAS  FAS-HT | 0.03 (0, 0.37) **  -  -  -  0.03 (0, 0.37) **  -  -  - | 0 (0, Inf) ^  -  -  -  0 (0, Inf) ^  -  -  - | | 10.6 (8.66, 12.6) *  -  -  -  10.6 (8.66, 12.6) *  -  -  - | |
| 1 - < 2 years |  |  |  | |  | |
| All NM-GFR (n=211)  NM-GFR ≤ 120 ml/min (n=211) | Schwartz  CKiD-U25  FAS  FAS-HT  Schwartz  CKiD-U25  FAS  FAS-HT | 0.11 (0.05, 0.25) *  0.38 (0.19, 0.78) **  -  -  0.11 (0.05, 0.25) *  0.38 (0.19, 0.78) **  -  - | 0.57 (0.30, 1.11)  0.69 (0.37, 1.31)  -  -  0.57 (0.30, 1.11)  0.69 (0.37, 1.31)  -  - | | 13.6 (12.3, 14.8) *  6.36 (5.10, 7.62) *  -  -  13.6 (12.3, 14.8) *  6.36 (5.10, 7.62) *  -  - | |
| 3 months - <2 years | | | |  |  |  |
| All NM-GFR (n=211)  NM-GFR ≤ 120 ml/min (n=211) | Schwartz  CKiD-U25  FAS  FAS-HT  Schwartz  CKiD-U25  FAS  FAS-HT | 0.15 (0.09, 0.24) *  -  -  -  0.15 (0.09, 0.24) *  -  -  - | 0.42 (0.26, 0.70) *  -  -  -  0.42 (0.26, 0.70) *  -  -  - | | 12.9 (11.8, 14.0) *  -  -  -  12.9 (11.8, 14.0) *  -  -  - | |
| ≥ 2 years | | | |  |  |  |
| All NM-GFR (n=1339)  NM-GFR ≤ 120 ml/min (n=1265) | Schwartz  CKiD-U25  FAS  FAS-HT  Schwartz  CKiD-U25  FAS  FAS-HT | 0.07 (0.05, 0.09) *  0.16 (0.12, 0.22) *  0.08 (0.06, 0.11) *  0.10 (0.07, 0.14) *  0.04 (0.03, 0.06) *  0.11 (0.08, 0.16) *  0.05 (0.04, 0.07) *  0.07 (0.05, 0.10) * | 0.42 (0.32, 0.55) *  0.55 (0.42, 0.71) *  0.43 (0.33, 0.56) *  0.51 (0.39, 0.66) *  0.34 (0.26, 0.44) *  0.47 (0.36, 0.62) *  0.35 (0.27, 0.46) *  0.42 (0.32, 0.55) * | | 20.3 (19.4, 21.2) *  15.6 (14.8, 16.5) *  20.0 (19.1, 20.9) *  19.0 (18.1, 19.9) *  19.4 (18.5, 20.2) *  14.4 (13.6, 15.3) *  18.8 (17.9, 19.6) *  17.6 (16.8, 18.5) * | |
| 2 – 12 years | | | |  |  |  |
| All NM-GFR (n=845)  NM-GFR ≤ 120 ml/min (n=830) | Schwartz  CKiD-U25  FAS  FAS-HT  Schwartz  CKiD-U25  FAS  FAS-HT | 0.05 (0.03, 0.08) *  0.23 (0.16, 0.35) *  0.11 (0.08, 0.17) *  0.15 (0.10, 0.22) *  0.04 (0.02, 0.06) *  0.20 (0.13, 0.30) *  0.09 (0.06, 0.14) *  0.13 (0.08, 0.19) * | 0.33 (0.24, 0.47) *  0.58 (0.42, 0.80) *  0.49 (0.35, 0.68) *  0.58 (0.42, 0.79) *  0.30 (0.22, 0.43) *  0.56 (0.40, 0.77) *  0.47 (0.34, 0.65) *  0.54 (0.39, 0.75) * | | 20.4 (19.6, 21.2) *  12.0 (11.2, 12.8) *  15.5 (14.7, 16.3) *  14.4 (13.6, 15.1) *  20.0 (19.2, 20.8) *  11.7 (10.9, 12.5) *  15.2 (14.4, 16.0) *  14.0 (13.2, 14.7) * | |
| > 12 years | | | |  |  |  |
| All NM-GFR (n=494)  NM-GFR ≤ 120 ml/min (n=435) | Schwartz  CKiD-U25  FAS  FAS-HT  Schwartz  CKiD-U25  FAS  FAS-HT | 0.10 (0.06, 0.17) *  0.08 (0.04, 0.13) *  0.04 (0.02, 0.07) *  0.05 (0.03, 0.08) *  0.03 (0.02, 0.06) *  0.02 (0.01, 0.05) *  0.01 (0, 0.02) *  0.01 (0.01, 0.03) * | 0.60 (0.39, 0.91) ***  0.49 (0.32, 0.76) *  0.33 (0.21, 0.52) *  0.40 (0.26, 0.62) *  0.39 (0.25, 0.63) *  0.33 (0.20, 0.53) *  0.18 (0.11, 0.31) *  0.24 (0.15, 0.40) * | | 20.1 (18.3, 21.9) *  21.8 (20.0, 23.6) *  27.6 (25.8, 29.4) *  27.0 (25.2, 28.8) *  18.2 (16.4, 20.1) *  19.7 (17.9, 21.5) *  25.6 (23.8, 27.5) *  24.6 (22.7, 26.4) * | |

NM-GFR, nuclear medicine glomerular filtration rate; EGFR, estimated glomerular filtration rate; P30, percentage of estimates within 30%; P10, percentage of estimates within 10%

* <0.001, ** <0.01, *** =0.012, ^ p-value could not be determined

**Supplementary Table 4 – Performance of different eGFR equations from NM-GFR results available before or from October 2021**

| Data | eGFR equation | P30 (%) (95% CI) | P10 (%) (95% CI) | | RMSE (95% CI) | | Bias (ml/min) (95% CI) |
| --- | --- | --- | --- | --- | --- | --- | --- |
| All ages | | | | | | | |
| All NM-GFR (n=1560)  NM-GFR before October 2021 (n=1170)  NM-GFR from October 2021 (n=390) | GROW-GFR  Schwartz  CKiD-U25  FAS  FAS-HT  GROW-GFR  Schwartz  CKiD-U25  FAS  FAS-HT  GROW-GFR  Schwartz  CKiD-U25  FAS  FAS-HT | 76.1 (73.9, 78.1)  46.4 (43.9, 48.8)  -  -  -  75.6 (73, 77.9)  53.2 (50.3, 56) -  -  -  77.7 (73.3, 81.5)  25.9 (21.8, 30.5)  -  -  - | 30.3 (28, 32.6)  18.3 (16.4, 20.3)  -  -  -  28.9 (26.4, 31.6)  21.5 (19.3, 24)  -  -  -  34.4 (29.8, 39.2)  8.5 (6.1, 11.7)  -  -  - | | 17.1 (16.5, 17.7)  27.7 (26.8, 28.7)  -  -  -  18 (17.3, 18.7)  24.3 (23.4, 25.4)  -  -  -  13.9 (13, 15)  36 (33.6, 38.7)  -  -  - | | -1.9 (-2.8, -1.1)  17.3 (16.2, 18.4)  -  -  -  -3.9 (-5, -2.9)  13.8 (12.7, 15)  -  -  -  4.1 (2.8, 5.4)  27.7 (25.5, 30)  -  -  - |
| < 3 months | | | |  | |  |  |
| All NM-GFR (n=10)  NM-GFR before October 2021 (n=7)  NM-GFR from October 2021 (n=3) | GROW-GFR  Schwartz  CKiD-U25  FAS  FAS-HT  GROW-GFR  Schwartz  CKiD-U25  FAS  FAS-HT  GROW-GFR  Schwartz  CKiD-U25  FAS  FAS-HT | 80 (49, 94.3)  10 (1.8, 40.4)  -  -  -  85.7 (48.7, 97.4) 14.3 (2.6, 51.3)  -  -  -  66.7 (20.8, 93.9)  0 (0, 56.2)  -  -  - | 30 (10.8, 60.3)  0 (0, 27.8)  -  -  -  42.9 (15.8, 75)  0 (0, 35.4)  -  -  -  0 (0, 56.2)  0 (0, 56.2)  -  -  - | | 4.3 (3.0, 7.9)  9.3 (6.4, 17)  -  -  -  4.6 (3, 10.2)  9.8 (6.3, 21.6)  -  -  -  3.6 (1.9, 22.7)  7.9 (4.1, 49.9)  -  -  - | | -2.6 (-4.9, -0.4)  8 (4.9, 11.1)  -  -  -  -2.4 (-5.5, 0.8)  8.2 (3.9, 12.5)  -  -  -  -3.2 (-5.5, -1)  7.6 (4.4, 10.8)  -  -  - |
| 3 months - <2 years | | | |  | |  |  |
| All NM-GFR (n=211)  NM-GFR before October 2021 (n=156)  NM-GFR from October 2021 (n=55) | GROW-GFR  Schwartz  CKiD-U25  FAS  FAS-HT  GROW-GFR  Schwartz  CKiD-U25*  FAS  FAS-HT  GROW-GFR  Schwartz  CKiD-U25*  FAS  FAS-HT | 74.4 (68.1, 79.8)  34.6 (28.5, 41.2)  60.1 (51.8, 67.9)  -  -  76.3 (69, 82.3)  41 (33.6, 48.9)  63.9 (55.8, 71.3)  -  -  69.1 (56, 79.7)  16.4 (8.9, 28.3)  29.8 (18.7, 44)  -  - | 26.5 (21, 32.9)  13.3 (9.3, 18.5)  20.3 (14.4, 27.8)  -  -  26.3 (20, 33.7)  16.7 (11.6, 23.3)  21.5 (15.6, 28.9)  -  -  27.3 (17.3, 40.2)  3.6 (1, 12.3)  17 (8.9, 30.1)  -  - | | 9.5 (8.7, 10.5)  16.4 (15, 18.1)  11.6 (10.4, 13.2)  -  -  9 (8.1, 10.1)  15.3 (13.7, 17.2)  10.9 (9.7, 12.3)  -  -  10.9 (9.1, 13.4)  19.2 (16.2, 23.7)  12.1 (10, 15.2)  -  - | | -1.9 (-3.2, -0.6)  11 (9.4, 12.7)  4.1 (2.3, 6)  -  -  -3.1 (-4.5, -1.8)  9.9 (8.1, 11.7)  3.2 (1.5, 4.9)  -  -  1.6 (-1.3, 4.5)  14.2 (10.7, 17.6)  9.3 (7.1, 11.5)  -  - |
| 2 – 12 years | | | |  | |  |  |
| All NM-GFR (n=845)  NM-GFR before October 2021  (n=638)  NM-GFR from October 2021 (n=207) | GROW-GFR  Schwartz  CKiD-U25  FAS  FAS-HT  GROW-GFR  Schwartz  CKiD-U25  FAS  FAS-HT  GROW-GFR  Schwartz  CKiD-U25  FAS  FAS-HT | 75.3 (72.3, 78.1)  43.8 (40.5, 47.2)  60.7 (57.4, 64)  52.9 (49.5, 56.2)  55.9 (52.5, 59.2)  74.5 (70.9, 77.7) 52.2 (48.3, 56.1)  68.3 (64.6, 71.8) 61.1 (57.3, 64.8) 63.5 (59.7, 67.1)  77.8 (71.6, 82.9) 17.9 (13.3, 23.7)  37.2 (30.9, 44) 27.5 (21.9, 34) 32.4 (26.4, 39) | 31 (28, 34.2)  16.7 (14.3, 19.4)  23.3 (20.6, 26.3)  21.2 (18.6, 24.1)  23.2 (20.5, 26.2)  29 (25.6, 32.6) 21 (18, 24.3)  27.7 (24.4, 31.3) 25.2 (22, 28.8) 27.3 (24, 30.9)  37.2 (30.9, 44) 3.4 (1.7, 6.8)  9.7 (6.3, 14.5) 8.7 (5.6, 13.3) 10.6 (7.1, 15.6) | | 16 (15.3, 16.8)  26 (24.8, 27.3)  20.4 (19.4, 21.4)  22.8 (21.8, 23.9)  22 (21, 23.1)  17.4 (16.5, 18.4) 22.3 (21.2, 23.6)  17.7 (16.8, 18.7) 19.4 (18.4, 20.5) 18.9 (17.9, 20)  10.7 (9.8, 11.9) 35 (31.9, 38.7)  27 (24.6, 29.8) 31.1 (28.3, 34.4) 29.5 (26.9, 32.6) | | -3.2 (-4.2, -2.1)  17.2 (15.9, 18.5)  8.9 (7.6, 10.1)  12.4 (11.1, 13.7)  11.2 (9.9, 12.5)  -5.6 (-6.9, -4.3) 13.3 (11.9, 14.7)  5 (3.7, 6.4) 8.3 (6.9, 9.6) 7.4 (6, 8.7)  4.4 (3.1, 5.7) 29.3 (26.7, 31.9)  20.7 (18.3, 23) 25 (22.5, 27.5) 23 (20.5, 25.5) |
| > 12 years | | | |  | |  |  |
| All NM-GFR (n=494)  NM-GFR before October 2021 (n=369)  NM-GFR from October 2021 (n=125) | GROW-GFR  Schwartz  CKiD-U25  FAS  FAS-HT  GROW-GFR  Schwartz  CKiD-U25  FAS  FAS-HT  GROW-GFR  Schwartz  CKiD-U25  FAS  FAS-HT | 78.1 (74.3, 81.6)  56.5 (52.1, 60.8)  53.6 (49.2, 58)  46.4 (42, 50.8)  48.6 (44.2, 53)  77 (72.4, 81) 60.7 (55.6, 65.6)  58.5 (53.5, 63.5)  49.6 (44.5, 54.7) 52 (46.9, 57.1)  81.6 (73.9, 87.4) 44 (35.6, 52.8)  39.2 (31.1, 48) 36.8 (28.9, 45.5) 38.4 (30.3, 47.2) | 30.6 (26.7, 34.8)  23.5 (20, 27.4)  21.1 (17.7, 24.9)  16.6 (13.6, 20.1)  18.6 (15.4, 22.3)  29.5 (25.1, 34.4) 24.9 (20.8, 29.6)  22.8 (18.8, 27.3)  19.5 (15.8, 23.9) 20.6 (16.8, 25)  33.6 (25.9, 42.3) 19.2 (13.3, 27)  16 (10.6, 23.4) 8 (4.4, 14.1) 12.8 (8, 19.8) | | 21 (19.7, 22.4)  33.9 (31.9, 36.2)  35.3 (33.2, 37.7)  39.6 (37.2, 42.2)  39.7 (37.4, 42.4)  21.6 (20.1, 23.3) 30.3 (28.2, 32.6)  32.6 (30.4, 35.2)  36.6 (34.1, 39.4) 37.5 (35, 40.4)  19 (16.9, 21.7) 43 (38.2, 49.1)  42.2 (37.6, 48.2) 47.3 (42.1, 54) 45.8 (40.7, 52.3) | | 0.2 (-1.7, 2)  20.3 (17.9, 22.7)  22 (19.6, 24.5)  27.8 (25.3, 30.3)  27.2 (24.6, 29.7)  -1.4 (-3.6, 0.8) 16.5 (13.9, 19.1)  18.7 (15.9, 21.4)  24.4 (21.6, 27.2) 24.4 (21.5, 27.3)  4.9 (1.7, 8.2) 31.6 (26.5, 36.7)  31.9 (27.1, 36.8) 37.8 (32.8, 42.8) 35.4 (30.3, 40.5) |

NM-GFR, nuclear medicine glomerular filtration rate; eGFR, estimated glomerular filtration rate; P30, percentage of estimates within 30%; P10, percentage of estimates within 10%; RMSE, residual mean standard error

* Children ≥1 year old
